# Whole-Body Physiologically Based Pharmacokinetic Modeling Framework for Tissue Target Engagement of CD3 Bispecific Antibodies

**DOI:** 10.3390/pharmaceutics17040500

**Published:** 2025-04-09

**Authors:** Monica E. Susilo, Stephan Schaller, Luis David Jiménez-Franco, Alexander Kulesza, Wilhelmus E. A. de Witte, Shang-Chiung Chen, C. Andrew Boswell, Danielle Mandikian, Chi-Chung Li

**Affiliations:** 1 Genentech, Inc., South San Francisco, CA 94080, USA; 2 ESQlabs GmbH, Am Sportplatz 7, 26683 Saterland, Germany

**Keywords:** T-cell engaging bispecific, TCB, PBPK, immune synapse, exposure–response, dose optimization

## Abstract

**Background**: T-cell-engaging bispecific (TCB) antibodies represent a promising therapy that utilizes T-cells to eliminate cancer cells independently of the major histocompatibility complex. Despite their success in hematologic cancers, challenges such as cytokine release syndrome (CRS), off-tumor toxicity, and resistance limit their efficacy in solid tumors. Optimizing biodistribution is key to overcoming these challenges. **Methods**: A physiologically based pharmacokinetic (PBPK) model was developed that incorporates T-cell transmigration, retention, receptor binding, receptor turnover, and cellular engagement. Preclinical biodistribution data were modeled using two TCB formats: one lacking tumor target binding and another with target arm binding, each with varying CD3 affinities in a transgenic tumor-bearing mouse model. **Results**: The PBPK model successfully described the distribution of activated T-cells and various TCB formats. It accurately predicted preclinical biodistribution patterns, demonstrating that higher CD3 affinity leads to faster clearance from the blood and increased accumulation in T-cell-rich organs, often reducing tumor exposure. Simulations of HER2-CD3 TCB doses (0.1 µg to 100 mg) revealed monotonic increases in synapse AUC within the tumor. A bell-shaped dose-Cmax relationship for synapse formation was observed, and Tmax was delayed at higher doses. Blood PK was a reasonable surrogate for tumor synapse at low doses but less predictive at higher doses. **Conclusions**: We developed a whole-body PBPK model to simulate the biodistribution of T-cells and TCB molecules. The insights from this model provide a comprehensive understanding of the factors affecting PK, synapse formation, and TCB activity, aiding in dose optimization and the design of effective therapeutic strategies.

## 1. Introduction

T-cell-engaging bispecific (TCB) antibodies represent an innovative class of immunotherapies, designed to harness endogenous T-cells to eliminate cancer or other diseased cells. TCB molecules function by simultaneous engaging CD3 receptors on T-cells and target receptors on diseased cells, forming an immune synapse. This synapse activates T-cells, facilitating targeted cytotoxicity independent of the major histocompatibility complex. The first wave of TCBs has been approved for several hematologic/non-solid malignancies, and many other compounds are currently under development [1]. Despite these successes, translating TCBs into effective therapies requires overcoming several challenges, including mitigating cytokine release syndrome (CRS), optimizing antitumor efficacy, minimizing on-target off-tumor toxicity, and addressing resistance mechanisms and durability [2]. Solid tumors present additional challenges due to their heterogeneous and immunosuppressive microenvironments, which hinder effective TCB delivery and therapeutic response [3].

A deeper understanding of TCB biodistribution and its impact on therapeutic outcomes is essential to overcoming current challenges. Physiologically based pharmacokinetic (PBPK) models are crucial tools that offer valuable insights into target engagement and synapse formation in the circulation, tumors, and tissues. They help elucidate the therapeutic index and identify potential target organs for safety concerns (e.g., on-target off-tumor toxicity). This understanding is particularly critical when tissue-specific target-mediated drug disposition (TMDD) makes extravasation a rate-limiting step for drug-target interactions [4].

TCBs exhibit dynamic target-mediated disposition, influenced by T-cell distribution kinetics, target expression/binding, and interactions with the neonatal Fc receptor (FcRn). Preclinical studies have demonstrated that TCB biodistribution is highly heterogeneous [5,6,7,8]. TCB often accumulates in T-cell-rich lymphoid tissues [9]. Notably, TCBs with higher CD3 affinity may have reduced tumor distribution due to preferential binding and clearance in these tissues [10]. PBPK models, alongside these experimental studies, help bridge the knowledge gap in the mechanistic understanding of T-cell redistribution and TCB synapse formation. The efficiency of pH-dependent binding to FcRn is another critical factor, impacting antibody recycling and systemic exposure [11]. Together, these insights underscore the need for an integrated approach to TCB development.

Several PBPK models have been developed to explore T-cell distribution and TCB pharmacokinetics (PK). Khot et al. [12] utilized a full PBPK model to characterize the whole-body distribution of exogenously administered T-cells in mice. More recently, Nikitich et al. [13] developed a PBPK model that describes endogenous T-cell homeostasis and the kinetics of exogenously administered T-cells in mice. From the perspective of TCB biodistribution, Zhang et al. [14] developed a full PBPK model to assess the effects of renal impairment on TCB PK and predict pediatric pharmacokinetics. Yoneyama et al. [15] used a simplified PBPK framework to predict immune synapse formation in tumor tissues. Their approach incorporated biodistribution using two-pore theory, extravasation of T-cells and hematologic cancer cells, TCB binding to targets, trimer formation, and competitive inhibition by shed targets. Beyond biodistribution, TCB downstream pharmacodynamic (PD) effects have been modeled using minimal PBPK-PD models and quantitative systems pharmacology (QSP) approaches. For instance, Jiang et al. [16] utilized a minimal PBPK-PD framework to describe blinatumomab PK, target cell killing, and cytokine release. Similarly, QSP approaches have been employed to study mosunetuzumab’s effects on B-cell, T-cell, and cytokine dynamics across circulation, lymphoid tissues, and tumors [17,18]. These QSP models supported clinical decision-making, including priming dose regimens to mitigate CRS and optimal doses for clinical efficacy.

Here, we develop a whole-body PBPK model that, for the first time, captures the biodistribution of both T-cells and TCB molecules. This model predicts complex (TCB-CD3 or TCB-target complex) and synapse (CD3-TCB-target synapse) formation across different organs. Building on an established PBPK platform [8], we extended it to include T-cell transmigration and retention, as outlined by Khot et al. [12]. We modeled biodistribution data from Mandikian et al. [9] for two TCB formats with differing CD3 affinities: one TCB lacking tumor target binding (glycoprotein D (gD)-CD3 TCB) and another TCB with a target arm binding to the receptor called the Human Epidermal growth factor Receptor 2 (HER2) (HER2/CD3 TCB) in a transgenic mouse model. The use of dual-tracer PK and biodistribution data enabled the differentiation between intact and internalized/catabolized TCB. Our model incorporated receptor binding, receptor turnover, and cellularity to simulate target antigen and CD3 complex formation, essential for assessing synapse formation—a critical driver of TCB-mediated on-target response.

Using a bottom-up and whole-body PBPK approach, this work examines the interplay between dosing, organ- or disease-specific properties (e.g., tumor location in lymphocyte-rich or lymphocyte-poor environments, effector-to-target (E–T) ratio), and TCB design parameters (e.g., dual binding affinities). This integrated approach offers a comprehensive understanding of the factors influencing synapse formation and TCB activity, guiding dose optimization and therapeutic design strategies.

## 2. Methods

### 2.1. T-Cell and TCB PBPK Model Development

We developed a whole-body PBPK model using the open-source software platform OSP-Suite [19,20,21], which includes PK-Sim [22,23] and MoBi [24]. The large molecule model, based on the two-pore formalism, served as the foundation. We extended this base model with MoBi to incorporate specific features relevant to our study.

**Figure 1:**
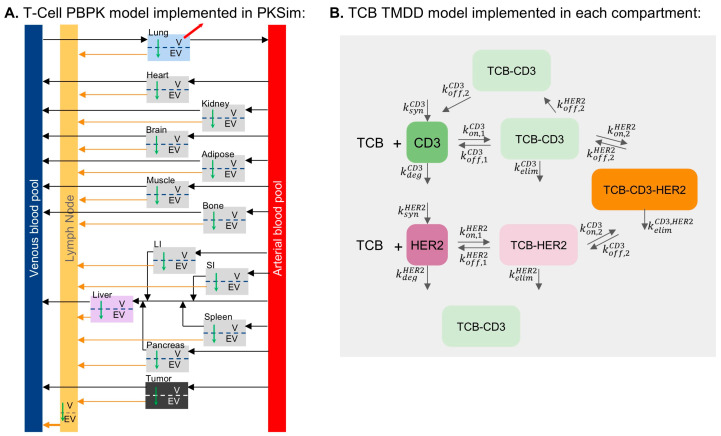
Schematic representation of (**A**) T-cell PBPK model and (**B**) T-cell-TCB-target cell synapse formation model. T-cell transmigration from vascular to extravascular space is indicated by the green arrow. k_deg_ and k_elim_ refer to the first-order degradation rates of the target and the TCB-target complexes, respectively; k_syn_ refers to the zero-order target synthesis rate constants; k_off_ refers to the first-order TCB-target dissociation rate constants; k_on_ refers to the second-order TCB-target dissociation rate constant.

The PBPK model integrates a murine whole-body physiological framework from PK-Sim, in line with the animal model studied and compound-specific characteristics (Figure 1). It includes a whole-body TCB distribution model, organ-specific HER2 protein expression, and a kinetic multi-state T-cell whole-body distribution model for T-cells (Figure 1A) defining the organ-specific CD3 disposition, CD3 recycling, HER2 turnover, and TMDD kinetics through CD3 and HER2. The TCB-CD3 complex, TCB HER2 complex, and CD3-TCB-HER2 synapse are represented in the model.

The default PBPK model structure was extended in MoBi to include a tumor module and a representative single lymph node organ. The tumor module was assigned a volume of 240 mg, which was the approximate total tumor burden reported in Mandikian et al. [9] and parametrized in the model following the approach of Niederalt et al. [22]. The lymph node included vascular, interstitial and intracellular compartments, and was assigned a volume of 4.04 mL/kg·BW [25], and vascular, interstitial, and intracellular fractions of 0.017, 0.53 and 0.453, respectively [26].

T-cell homeostasis was modeled based on kinetic data from exogenously activated and administered T-cells in mice [12], adopting the assumption that elimination occurs in the lung interstitial space and omitting T-cell proliferation over a short time scale. Model fitting to this dataset (see Supplementary Figure S2) allowed for the estimation of the transmigration rate constants and tissue retention factors of T-cells. It was assumed that activated T-cells exhibit a tenfold higher transmigration rate than naive T-cells, while retaining identical retention factors [27].

The complex and synapse dynamic model extension was implemented in MoBi (Figure 1B). For HER2 TMDD, the synthesis and degradation rates for free and bound HER2 were assumed to be identical [28]. For CD3 TMDD, the free and bound CD3 recycling was parameterized to achieve a steady state where the membrane CD3 levels are twice the internalized amount. This was defined using first-order rate constants ($k_{rec}^{CD3}$) and ($k_{int}^{CD3}$ = 0.5$k_{rec}^{CD3}$).

The synapse formation is described by two reversible binding processes: CD3-TCB complex binding to HER2 and HER2-TCB complex to CD3, both of which follow mass action kinetics. An avidity factor was introduced to scale $k_{off,2}^{CD3}$ and $k_{off,2}^{HER2}$ [29]. As part of the TMDD network, internalization of the CD3-TCB-HER2 synapse has been incorporated, consistent with Schropp et al. [28]. This accounts for the high stability of the complex, whereby a receptor is “stripped” from the non-internalizing target cell, similar to trogocytosis of physiological synapses [30].

The avidity factor and internalization rate constant govern total CD3-mediated internalization at the T-cell synapse. While their interplay was not explicitly explored, the internalization rate constant, fit to in vivo data, was 0.3 relative to dimer complexes and the avidity factor was set to 100—consistent with reported in vivo values and the stable functional avidity of CD8 T-cells [29]. Moreover, the low synapse dissociation rate constant effectively renders the formation irreversible, aligning with the high molecular density and stability of the immunological synapse. Given the broad exploration of dose and effector-to-target ratios, uncertainty in these assumptions is unlikely to impact the study’s conclusions.

Parameterization was guided by literature values and iteratively refined throughout model development and evaluation in MoBi, following three calibration stages (Supplementary Figure S1):

1. Calibration of T-cell distribution parameters. This stage is described in Section 2.2.
2. Calibration of parameters for the distribution of gD-only and gD-CD3 TCB. This stage is described in Section 2.3.
3. Calibration of parameters for the distribution HER2-CD3 TCB. This stage is described in Section 2.3.
4. The complete set of parameters are summarized in Supplementary Table S1.

### 2.2. Calibration of T-Cell Distribution

To describe the T-cell distribution data from Khot et al. [12], two organ-specific parameters in the model were calibrated: T-cell transmigration rate and T-cell retention factor. Supplementary Figure S2 shows that the model simulation using these calibrated parameters captures the distribution of exogenously administered activated T-cells, with the exception of the lymph node compartment. The lymph node transmigration rate and retention rate were not calibrated using this dataset, as measurements were only available from inguinal lymph nodes, which do not align with the lumped lymph node compartment represented in the model. A lumped lymph node compartment was chosen because the TCB distribution data used for calibration (Section 2.3) consisted of aggregate measured lymph samples. In this case, the lymph node tissue distribution was assumed to be similar to that of the spleen [31].

The T-cell distribution data from Khot et al. [12] describes activated T-cells, rather than resting T-cells. To evaluate whether the model can also capture the steady-state distribution of resting T-cells, we simulated their distribution using a migration rate that is ten times lower than the calibrated value for activated T-cells [17,27,32]. The resulting resting T-cell tissue distribution aligned with previously published observed data (Supplementary Figure S3).

### 2.3. Calibration of gD/CD3 TCB and HER2/CD3 TCB

The biodistribution data from Mandikian et al. [9] for gD-CD3 TCB and HER2-CD3 TCB in a transgenic mouse model was used to calibrate the TCB PBPK model. Prior to calibration of the TCBs, the PK-Sim’s base PBPK model parameters were modified to fit the distribution data of gD antibody, which is an antibody without target or CD3 binding, in the same transgenic mouse model as [9]. The mouse tissue physiological parameters (i.e., vascular and interstitial volume fractions) in PK-Sim were adjusted to available physiological data of the specific mouse model used [26]. Other key parameters, such as FcRn affinity and the residual blood fraction, which accounts for the partial loss of blood from tissue samples, were also adjusted for each tissue. The parameters that reasonably captured the observed gD antibody distribution data can be found in Supplementary Table S1.

In Mandikian et al. [9], TCB was labeled with residualizing (^111^In) and non-residualizing (^125^I) isotopes. The non-residualizing radiolabel measurements were used to calibrate organ-specific parameters, reflecting intact TCB in tissues. In contrast, residualizing radiolabel measurements calibrated systemic parameters, avoiding the influence of radiolabel efflux after TCB catabolism.

Simulation scenarios for model calibration were defined according to the experimental setup in [9]. The CD3-related parameters (k_syn_, k_deg_, k_elim_, k_on_, k_off_) and HER2 parameters (k_syn_, k_deg_, k_elim_, k_on_, k_off_) were fitted in a hybrid, stepwise approach, using both manual adjustments and gradient-based parameter identification algorithms (Levenberg–Marquardt) included in the OSP Suite.

Binding and synapse formation kinetics were informed by in vitro binding data, employing several assumptions without further calibration against experimental data:

- Synapse internalization through trogocytosis was set to be slower (1/3) than internalization of TCB receptor complexes due to the assumed additional energy required to “strip” the receptor from the non-internalizing cell. Bigger differences in this factor led to little differentiation between the HER2-CD3L and HER2/CD3H scenarios.
- An avidity factor (×0.01) was introduced to account for increased binding affinity due to proximity between T-cells and tumor cells during synapse formation, enhancing the likelihood of receptor rebinding. This had a low impact on TCB PK but a high impact on synapse formation.

### 2.4. Model-Based Evaluations and Analyses

All figures shown in the manuscript are defined by a set of simulation parameters defined in Supplementary Table S1. For any simulation, the model was set with CD3 concentrations as described in the Supplementary Materials. TCB was administered as a bolus, according to the relevant experimental dosing protocol (0.1 ug to 100 mg), and, in some cases, run for 5000 h to accurately determine the area under the curve (AUC). Fractions of residual blood for sampling for all organs were adjusted to fit the T-cell data [12], and the same values were used for TCB.

#### 2.4.1. Evaluation of TCB Distribution

The distribution of TCBs with different binding affinity in the whole blood, T-cell-rich organs, tumor, and other organs at 72 h post-dose was used to fit the model parameters [9]. TCBs were administered as a 0.5 mg/kg IV bolus labeled with a mixture of 5 uCi of a residualizing (^111^In) and 5uCi of a non-residualizing (^125^I) radiolabel, according to Supplementary Table S1.

#### 2.4.2. Model-Based Evaluation of Binding Kinetics and Synapse Formation

To evaluate the kinetic behavior of TCB, complex and synapse across different tissues, a dose of 0.01 mg TCB for a 20 g mouse was used. We further evaluated the effect of dose level on the PK of TCB, the TCB-CD3 complex, and the synapse in blood and tumor tissues across all simulated dose levels ranging from 10 µg to 100 mg. A similar setup was used to evaluate HER2 and CD3 receptor occupancy over time.

The relationship between TCB tissue levels and synapse formation was evaluated under various E–T ratios by varying the transmigration rate of T-cells into the tumor between 0.01 and 100, while keeping the target cell number in the tumor constant. 

### 2.5. Software Used

The base PBPK models for TCBs and T-cell biodistribution were developed using the OSP Suite (i.e., PK-Sim^®^ and MoBi^®^), version 11.3. For model calibration and simulation scenarios, we utilized the open-source esqlabsR framework with R version 3.6.3. This framework is compatible with R and allows for model (re)parameterization and the setup of simulation scenarios using modeling files exported from PK-Sim^®^ and MoBi^®^. Plots were generated using the ggplot2 package in R. Simulation scenario settings, including parameter values and doses, were defined within separate .csv files (Supplementary Table S1) and loaded into R. All necessary simulation scenarios and model files are available as Supplementary Materials.

## 3. Results

### 3.1. PBPK Model Simulations of Distribution of T-Cell, gD-CD3 TCB, and HER2-CD3 TCB

**Figure 2:**
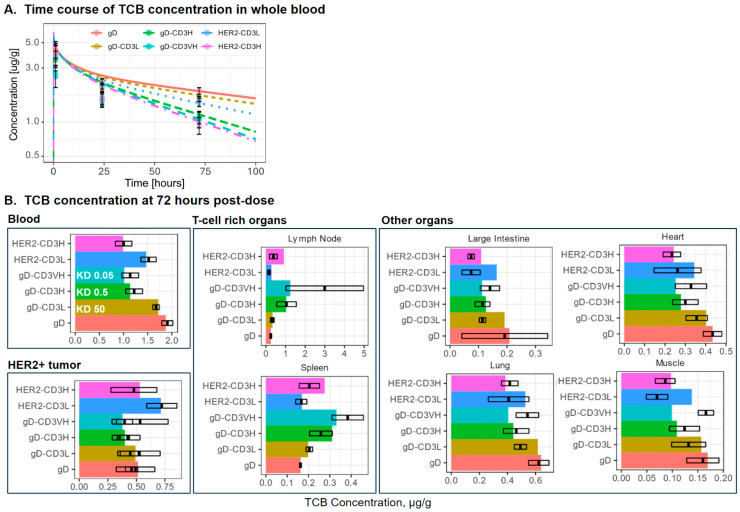
The model captures the distribution of TCBs with different binding affinity in the whole blood, T-cell-rich organs, tumor, and other organs at 72 h post-dose. TCBs were administered as a 0.5 mg/kg IV bolus labeled with a mixture of 5 uCi of a residualizing (^111^In) and 5uCi of a non-residualizing (^125^I) radiolabel. Blood data correspond to measurements with a residualizing radiolabel, while organ data were acquired with a non-residualizing radiolabel. Colored bars represent model simulation, while the black box plots represent the mean and standard error of the observed TCB concentration from Mandikian et al. [9].

The T-cell PBPK model captures the distribution of exogenously administered activated T-cells data (Supplementary Figure S2) reported in Khot et al. 2019 [12] and aligns with the steady-state distribution of resting T-cells reported by Rubin et al. [31] (Supplementary Figure S3). Figure 2A shows the model simulated and observed TCB concentration–time profiles in blood for all tested formats. The gD antibody displays a bi-exponential decline, consistent with typical antibody pharmacokinetics and its PK is well captured by the model. Blood distribution and clearance increase with binding affinity in the order of gD-CD3L, HER2-CD3L, gD-CD3H, gD-CD3 VH, and HER2-CD3H, as indicated by decreasing concentrations measured 72 h post-dose. The model captures this trend at 72 h but over-estimates the peak concentrations in blood 1 h post-dose for the CD3-binders. The reason for the CD3-binding effect on blood PK at early time points is unclear and might be related to transient T-cell responses.

Figure 2B presents the model-simulated and observed TCB concentrations across various organs, evaluating the impact of T-cell distribution on TCB biodistribution. The distribution of T-cells across organs influences the biodistribution of both gD-CD3 and HER2-CD3 TCB, as observed in Mandikian et al. [9]. The model captures the effect of organ T-cell density on antibody biodistribution based on experimental observations at 72 h (Figure 2B), except in the case of the gD-CD3VH. While the model captures the gD-CD3VH distribution trends in the blood, tumor, and T-cell-rich organs, it underestimates the gD-CD3VH concentration in other organs. Specifically, the model predicts a decrease in gD-CD3 concentration with increasing affinity. In contrast, the observed data show a decrease in gD-CD3 concentration from low to high CD3 affinity, followed by an increase at very high CD3 affinity. The observed trend for gD-CD3VH may be attributed to the higher recruitment into the T-cell-rich organs due to T-cell activation, which is not accounted for in the model. Higher CD3 affinity in gD-CD3 TCB reduced the blood PK, increased exposure to T-cell-rich organs (e.g., lymph node, spleen), and reduced or retained exposure in other organs. In the HER2-CD3 TCB case, a similar trend to the gD-CD3 TCB was observed with higher CD3 binding affinity. The tumor exposure of HER2-CD3 TCB is reduced in higher CD3 binding affinity cases. The presence of the HER2 target on the HER2-CD3 TCB reduced the plasma and lymph node distribution compared to gD/CD3 TCB, plausibly due to more mAbs being redirected into the HER2+ tumor compartment. The agreement of model simulations with the observed T-cell and antibody distribution established the foundation for evaluating synapse formation in different organs.

### 3.2. The Kinetics of TCB-CD3-HER2 Synapse Is Dose-Dependent

**Figure 3:**
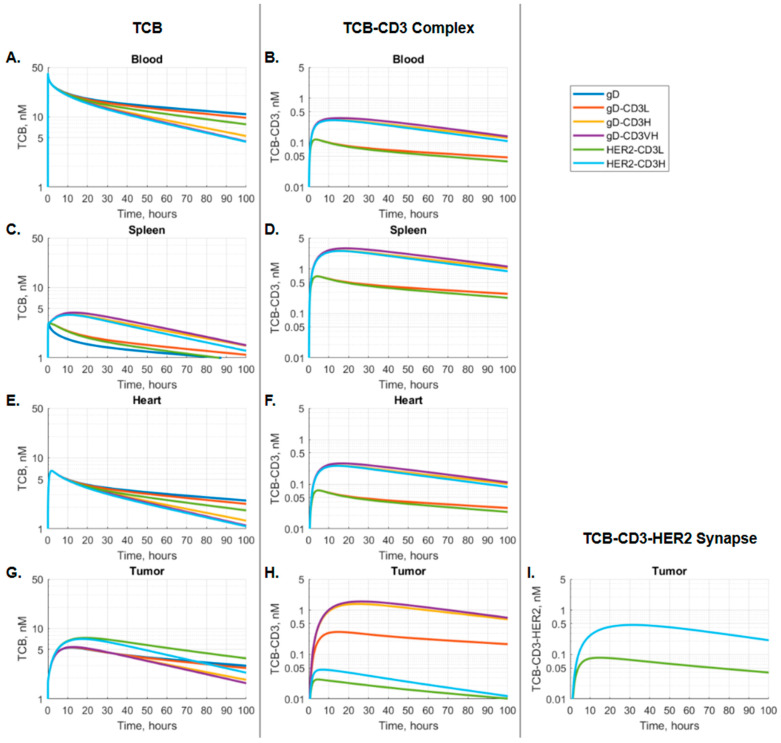
The concentration of (**A**,**C**,**E**,**G**) TCB, (**B**,**D**,**F**,**H**) TCB-CD3 complex, and (**I**) TCB-CD3-HER2 synapse, over time in blood, T-cell rich organs (e.g., spleen), other organs (e.g., heart), and tumor. TCBs with varying CD3 and HER2 affinities were administered as a 0.5 mg/kg IV bolus, equivalent to a mass dose of 0.01 mg TCB for a 20 g mouse.

Figure 3 illustrates the kinetic profiles of TCB and TCB-CD3 complex concentrations over time in representative organs, providing insight into kinetic behavior across different tissues. A single dose of TCB leads to the TCB-CD3 complex formation in all compartments and the TCB-CD3-HER2 synapse formation in the tumor (Figure 3). As shown in Figure 3A, blood distribution and clearance rates increase in the order of gD-CD3L, HER2-CD3L, gD-CD3H, gD-CD3 VH, and HER2-CD3H, as indicated by the slopes of the concentration-time profiles. The TCB-CD3 complex concentrations reflect binding affinity—TCBs with higher CD3 affinity (gD-CD3 VH, gD-CD3H, and HER2-CD3H) exhibit higher TCB-CD3 complex concentrations compared to those with lower CD3 affinity (gD-CD3L, HER2-CD3L), as expected. This trend is consistent across blood and tissues, with the exception of the tumor, where TCB-CD3 complex formation is further influenced by the formation of TCB-CD3-HER2 synapses (Figure 3H,I).

Notable PK differences in TCB and TCB-CD3 complexes are observed across blood, T-cell-rich organs (e.g., spleen), and non-T-cell-rich organs (e.g., heart), as depicted in Figure 3A–H.

Simulated blood TCB concentration-time profiles show an immediate peak in TCB blood concentrations. This is followed by a delayed peak in TCB-CD3 complex concentrations, driven by the binding on/off kinetics (Figure 3A,B).

In T-cell-rich organs, such as the spleen, higher-affinity TCBs tend to accumulate at higher concentrations, both as free TCB and as TCB-CD3 complexes. This process is characterized by slower accumulation and a delayed Tmax (Figure 3C,D). In non-T-cell-rich organs, such as the heart, TCB and TCB-CD3 complex concentrations, as well as Tmax values, closely mirror the PK profiles observed in the blood (Figure 3A,B,E,F).

In tumor tissues, TCB PK consistently peaked later than in the circulating blood. Notably, HER2-targeting TCBs (HER2-CD3H and HER2-CD3L) achieved higher tumor tissue concentrations than gD-targeting TCBs, highlighting the critical role of HER2 binding in driving tumor distribution of TCB. Among gD-targeting molecules, the higher-affinity gD-CD3 variants (gD-CD3H, gD-CD3VH) formed the most TCB-CD3 complexes, surpassing the HER2-targeting TCBs (HER2-CD3L, HER2-CD3H). Between the HER2 TCB variants, the higher-affinity HER2-CD3H showed increased peak synapse formation and a delayed Tmax for synapse formation (Figure 3G–I).

**Figure 4:**
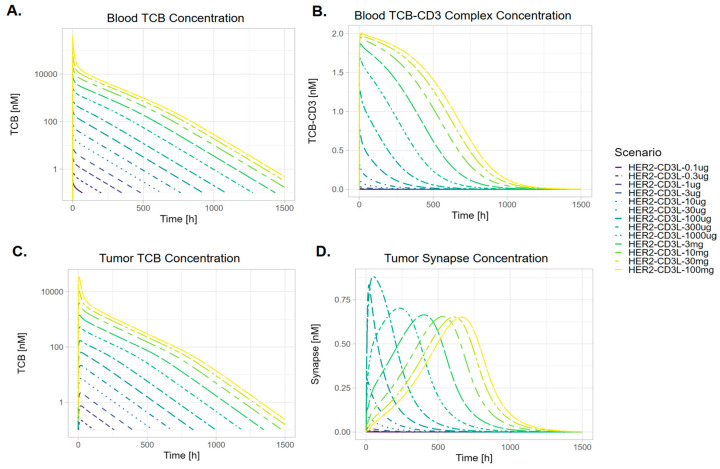
Concentration-time profiles in blood for (**A**) TCB, (**B**) TCB-CD3 complex, and in tumor for (**C**) TCB and (**D**) synapse, for low-CD3-affinity TCB (HER2-CD3L) following IV administration at selected simulated dose levels (0.1 µg to 100 mg).

**Figure 5:**
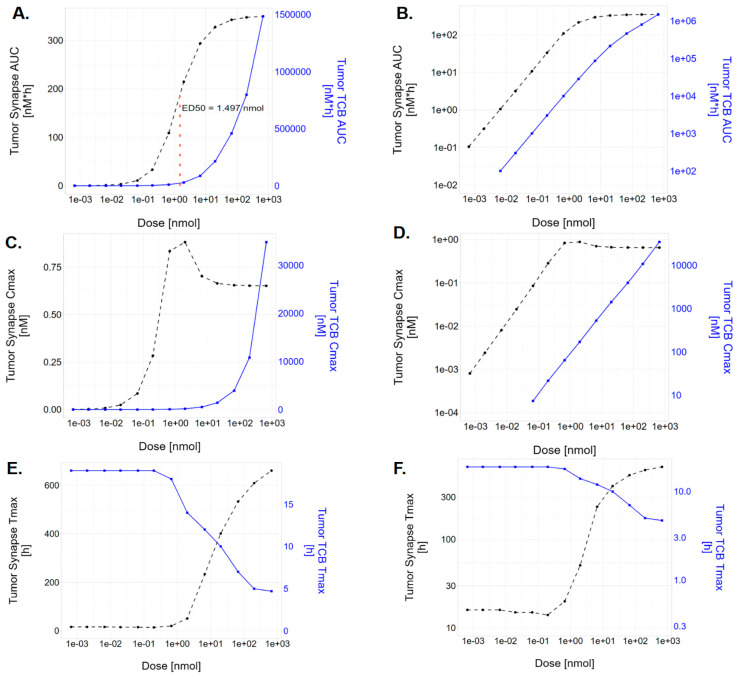
Dose dependency of derived PK metrics of TCB (blue lines) and synapse (black dashed lines) in the tumor following IV TCB administrations: (**A**,**B**) area under the curve (AUC) from time of dose to infinity, (**C**,**D**) maximum concentration (Cmax), and (**E**,**F**) time to maximum concentration (Tmax). In all simulations, a single dose was applied, ranging from 0.1 ug to 100 mg. (**A**,**C**,**E**) use a linear scale on the vertical axis, while (**B**,**D**,**F**) use a logarithmic scale on the vertical axis.

When the effect of dose on the PK of TCB, TCB-CD3 complex, and synapse in blood and tumor tissues was evaluated (Figure 4 and Figure 5), it was observed that the PK of TCB in blood and tumor increased proportionally across all simulated dose levels (10 µg to 100 mg). In contrast, synapse PK demonstrated dose-dependent nonlinearity. At low doses (0.1 µg to 100 µg), synapse Cmax increased with dose. However, at higher doses (300 µg to 100 mg), Cmax decreased, with a further delay in Tmax (Figure 4D and Figure 5E,F). This pattern resulted in a slight bell-shaped synapse Cmax-dose curve (Figure 5C,D). At the highest doses, synapse Cmax stabilized at similar values (Figure 4D and Figure 5C,D). In comparison, synapse AUC increased monotonically with dose and plateaued at higher dose levels (Figure 5A,B).

### 3.3. The CD3 Arm Affinity Influences the Kinetics of TCB-CD3-HER2 Synapse

**Figure 6:**
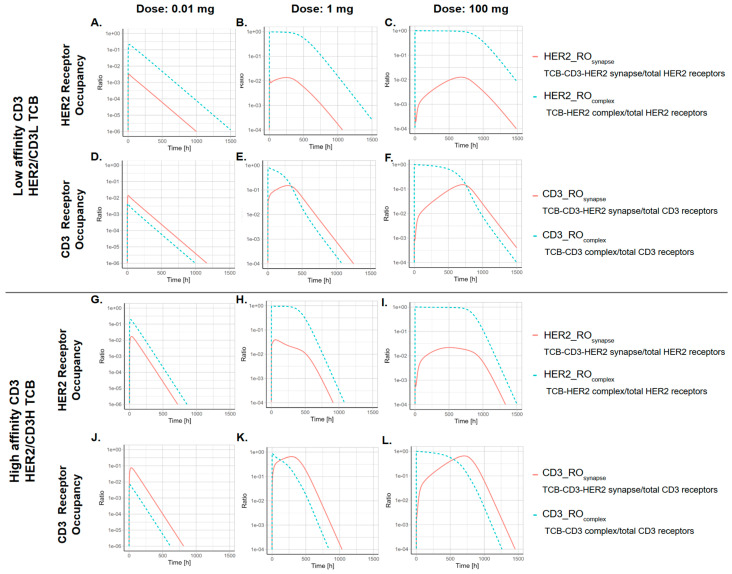
For the low CD3 affinity TCB, (**A**,**B**,**C**) HER2 receptor occupancy (1 = 100% occupancy) over time in the tumor by the synapse (HER2_RO_synapse_) and by the TCB-HER complex (HER2_RO_complex_), (**D**,**E**,**F**) CD3 receptor occupancy by the synapse (CD3_RO_synapse_) and by the TCB-CD3 complex (CD3_RO_complex_). (**G**–**L**) The receptor occupancy for the high CD3 affinity TCB. Simulation from three dose levels (0.01 mg, 1 mg, 100 mg) are shown.

Figure 6 shows HER2 receptor occupancy in the tumor by the synapse (HER2_RO_Synapse_) and by the TCB-HER complex (HER2_RO_TCB_Complex_), as well as CD3 receptor occupancy by the synapse (CD3_RO_Synapse_) and by the TCB-CD3 complex (CD3_RO_TCB_Complex_), at three dose levels (0.01 mg, 1 mg, 100 mg), comparing two TCBs with different CD3 affinities (HER2-CD3L vs. HER2-CD3H).

At low doses (0.01 mg), tumor HER2 receptors and CD3 receptors are not saturated. Therefore, synapse formation kinetics follows TCB concentration kinetics, with higher initial synapses formed and then faster clearance for the higher CD3 affinity (HER2-CD3H) antibody. In this case, the majority of CD3 receptors are occupied by the synapse. Additionally, at the low dose level (0.01 mg), the temporal profile of HER2_RO_Synapse_ mirrors that of HER2_RO_TCB_Complex_, as neither TCB binding to HER2 nor CD3 receptors are saturated. Higher CD3 affinity leads to higher overall synapse receptor occupancy, as expected.

At higher dose levels (1 mg and 100 mg), the tumor HER2 receptors and CD3 receptors are initially saturated. When receptors are saturated, there is low synapse occupancy, but as the TCB concentration decreases, the synapse receptor occupancy increases. As HER2 and CD3 receptors are initially saturated by TCB complexes, synapse formation is restricted, resulting in a flattened temporal curve with delayed Tmax values for both HER2_RO_Synapse_ and CD3_RO_Synapse_.

### 3.4. The Kinetics of TCB-HER2-CD3 Synapse Is Dependent on the Ratio Between the Effector-to-Target Cell Ratio

**Figure 7:**
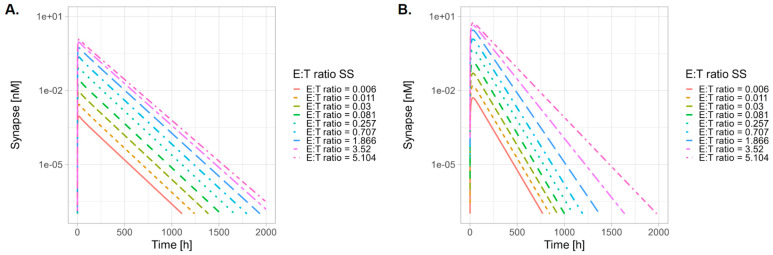
Tumor concentrations of TCB-CD3-HER2 synapse over time, simulated at various effect-to-target (E–T) ratios for HER2-TCB with (**A**) low CD3 affinity (HER2-CD3L) and (**B**) high CD3 affinity (HER2-CD3H). Administered dose of 0.5 mg/kg IV bolus, equivalent to a mass dose of 0.01 mg TCB for a 20g mouse.

The effector-to-target (E–T) ratio was varied by altering the transmigration rate of T-cells into the tumor (0.01 to 100) while keeping the target cell number in the tumor constant. This variation resulted in baseline E–T ratios ranging from 0.006 to 5.104. The results indicate that higher synapse concentrations are associated with both a higher E–T ratio and greater CD3 affinity of the HER2 TCB molecule (Figure 7). For the high CD3 affinity TCB, the higher E–T ratio was associated with reduced synapse clearance, as indicated by the reduced slope in the synapse PK profile post peak (Figure 7B).

## 4. Discussion

The clinical activity of TCB molecules depends on their mechanism of action, which involves simultaneous binding to effector T-cells and target cells to form immune synapses. In this work, we present an integrated understanding of immune synapse PK across organs, using a full PBPK model to characterize the biodistribution of both TCB antibodies and T-cells. Additionally, we incorporate mechanistic modeling of the multi-step binding kinetics for synapse formation.

Our model successfully recapitulates preclinical experimental data and captures observed trends in TCB constructs with varying targets (gD-CD3, HER2-CD3) and CD3 affinities. To our knowledge, this is the first integrated platform enabling the characterization and prediction of key pharmacological drivers (e.g., synapse formation) for on-target efficacy and safety following TCB administrations. It accounts for practical factors such as target expression, receptor turnover, TCB design elements (e.g., binding affinity/avidity), and disease characteristics (e.g., E–T ratio).

### 4.1. CD3 Affinity on Pharmacological Drivers of Efficacy vs. On-Target Safety

The impact of CD3 affinity has been a critical factor in the antibody engineering design of TCB molecules. Higher CD3 affinity is typically associated with increased cytotoxicity potency and tumor-killing efficiency in vitro [33] as well as stronger exposure–response relationships in clinical settings. However, elevated CD3 affinity also drives rapid acute T-cell activation, which can lead to a higher incidence of clinical toxicities, such as CRS [33].

In this study, we introduced an additional dimension for optimizing the benefit-risk profile of TCB molecules: tumor distribution efficiency. Using dynamic systems modeling of T-cell and target cell distributions alongside TCB PK, distribution, and binding kinetics, we showed that reduced CD3 affinity improves tumor-to-blood partitioning. Specifically, low-affinity CD3 TCBs (HER2-CD3L) achieved higher tumor TCB concentrations than high-affinity CD3 TCBs (HER2-CD3H). Despite this, the model predicts higher and delayed synapse peaks for HER2-CD3H, suggesting more effective synapse formation with high-CD3-affinity TCBs. These findings highlight tumor distribution efficiency and binding kinetics as critical factors for enhancing pharmacological efficacy at the tumor site.

Systemic TCB concentrations, often associated with clinical safety risks (e.g., on-target/off-tumor toxicities in solid tumors), are correlated with higher CD3 binding affinity [34]. High-affinity CD3 TCBs in this study led to increased systemic TCB-CD3 complex formation, suggesting higher synapse concentrations in cases of target expression in circulation of off-tumor organs. These findings underscore the importance of balancing tumor efficacy with systemic safety risks.

### 4.2. Blood PK Metric as Surrogate for Dose–Response Characterization

In this study, we evaluated multiple metrics to investigate the relationship between dose, TCB PK, complex PK, and synapse PK across tissues using the PBPK model. For synapse PK, we further analyzed the dose–PK relationship using various metrics, including AUC, Cmax, and Tmax in both blood and tumor compartments.

At sub-saturating levels for CD3 and target binding, blood TCB PK exposure metrics serve as a reasonable surrogate for target engagement in the tumor for exposure–response analyses. This is supported by the correlation between blood PK and tumor PK (Figure 4A,C) as well as between tumor PK and synapse concentration (Figure 5B,D). The PK of all relevant moieties is generally dose-proportional, making blood PK a practical metric to characterize clinical effects. This is particularly relevant since TCB molecules have been shown to be effective at lower receptor occupancy levels due to their mechanism of action as conditional agonists [35,36]. PK surrogate metrics have been utilized for exposure–response assessment for clinical efficacy and CRS endpoints in approved TCBs (Table 3 of Radtke et al. [37] summarizes the exposure metrics used). For instance, in BCMA-CD3 TCB molecules to treat Multiple Myeloma [38,39,40], the PK metrics were statistically significant (Cavg for efficacy and Cmax for CRS). Similarly, in CD20-CD3 TCB molecules to treat Non-Hodgkin’s Lymphoma [41,42], CD20 RO% empirically derived from blood PK were statistically significant (AUC for efficacy, peak for CRS).

At saturating synapse levels, as the TCB AUC and Cmax continue to increase (Figure 5A–D), synapse AUC plateaus, and the synapse Cmax first peaks, then declines, and finally plateaus at higher doses, whereas the TCB Cmax keeps rising. This synapse metric trend can be explained as follows: At a low dose range, there is insufficient TCB concentration to accumulate the synapse, emphasizing the TCB disposition governing the kinetics. At a high dose range, right after dosing, the TCB temporarily saturates the CD3 and HER2 receptors, which slows the further formation of synapses. However, as the TCB clears and the concentration is sufficiently low, again, more synapses can be formed. The high concentration also allows the synapse concentration to be maintained longer, causing the AUC of the synapse to continue increasing.

At higher doses beyond the AUC and peak concentration metrics, the synapse accumulation in the tumor occurs more slowly, resulting in a delayed peak (Figure 4D) compared to the blood PK (Figure 4A,C). The timing of the peak synapse may affect the onset of CRS or shift the peak to a time point where cytokine release is less sensitive to stimulation.

Previous in silico studies have demonstrated that the relationship between the synapse formation TCB concentration can form a bell shape [43], thus suggesting that if the TCB dose is too high, this may result in lower synapse formations, which, subsequently, have the potential to affect both the tumor-killing effect and on-target adverse event of cytokine release syndrome. The bell-shaped curve of synapse formation has been theoretically demonstrated [43] and observed in in vitro systems; however, its clinical significance in vivo remains unclear due to the dynamic changes in TCB concentrations and the varying E–T ratios across organs and over the course of treatment. Our work therefore employs a holistic and integrated theoretical framework to evaluate the significance of the bell-shaped dose–response relationship in an in vivo context, accounting for cell–cell interaction, cell dynamics, trafficking, and receptor turnover. Despite the bell-shaped relationship between synapse and TCB concentration observed in vitro, our results indicate that the synapse AUC (Figure 5A,B) monotonically increases with dose and appears to have a sigmoid relationship. On the other hand, the peak synapse (Figure 5B) appears to indeed feature some bell-shaped relationship which does not decline to zero but instead plateaus at some level below its maximum. It is possible that the specific molecule properties of this study do not allow the bell-shaped relationship within the simulated dose range.

From an efficacy perspective, aggressive diseases may be more sensitive to delayed synapse onset kinetics, making a bell-shaped dose response more relevant in such clinical contexts. Additionally, synapse Cmax in the blood could serve as a marker for clinical CRS risk. Derived CD20 RO has previously been reported by mosunetuzumab as a significant driver for clinical CRS [35]. As demonstrated, under subsaturating conditions, target_RO_Complex_ mirrors that of target_RO_Synapse_, rendering target RO a reasonable surrogate metric for exposure–response characterizations.

### 4.3. Tumor E–T Ratio

The variability in the tumor E–T ratio can be attributed to the level of tumor burden or the extent of lymphocyte infiltration into the tumor. However, even within the same indication there is a high variability of E–T ratio [18].

The E–T ratio is a critical factor in determining the potency of TCB molecules, as established in vitro [44]. In this study, we simulate the impact of the E–T ratio on synapse formation in an in vivo setting, using HER TCB tool molecules with varying CD3 affinity. Our findings indicate that an increased E–T ratio enhances synapse PK, leading to higher peak synapse levels (Figure 7). Notably, for high CD3 affinity molecules, there is a change in the elimination phase of the synapse concentration–time curve, suggesting nonlinear behavior as the E–T ratio increases (Figure 7B).

Despite the success of TCB treatments in hematological malignancies, their application in solid tumors remains challenging, with only one molecule approved for uveal melanoma [45]. The tumor microenvironment and cancer antigen expression are pivotal in determining therapeutic potential via effective synapse formation. Cold tumors, characterized by poor T-cell infiltration and low E–T ratios, may limit the therapeutic effectiveness of TCB molecules. Thus, to improve therapeutic outcomes, the future of TCB development will likely focus on combination strategies to revitalize the immune context [46], such as checkpoint inhibitors, co-stimulatory molecules, cytokine therapies, and antibody–drug conjugates. Such combinations can also shift the potency and effectiveness of TCB through more effective synapse formation.

Our PBPK framework offers a theoretical platform to simulate therapeutic potential across diseases with varying E–T ratios, providing valuable insights into treatment strategies.

### 4.4. Future Directions

Our PBPK model was calibrated using a mouse model with HER2 expression confined to the tumor. This exclusive target expression may not reflect other preclinical or clinical scenarios. Extending the model to include normal tissue expression unique to the modeled TCB and species could be valuable for cross-species translations. Additionally, further validation using other preclinical TCB distribution data will strengthen the model’s robustness. In addition, more mechanistic immune synapse modeling, such as receptor clustering, membrane restriction, and mobility, has not been considered, as the focus was on biodistribution and its importance for synapse disposition kinetics.

While the model primarily focuses on PK, biodistribution, and target engagement through synapse formation, the relationship between synapse formation and TCB activity is outside the scope of this work. However, it serves as a foundational framework for future modeling efforts linking synapse dynamics to TCB activity. Incorporating different T-cell functionalities, such as activation, proliferation, and cytotoxicity, could enhance its clinical applicability. Despite its limitations, this framework lays the groundwork for understanding synapse dynamics and guiding future strategies in TCB development. Future opportunities include integrating the PBPK model with QSP modeling describing tumor cell killing and cytokine production, thereby linking tissue-level target engagement with clinical efficacy and CRS predictions.

## 5. Conclusions

This study presents a comprehensive whole-body PBPK model to understand the biodistribution and kinetics of TCB antibodies. Our model integrates the distribution dynamics of T-cells and TCB molecules, providing critical insights into the formation of complexes and immune synapses across various tissues. This approach highlights the interplay between dosing strategies, organ-specific properties, and TCB design parameters, such as CD3 affinity, which significantly influence synapse formation and therapeutic outcomes.

The PBPK model elucidates the kinetics and dose-dependency of TCB PK, complex (TCB-CD3, TCB-target) and synapse formation in blood, T-cell rich organs (e.g., spleen and lymph node), tumor, and other organs. Overall, this model enhances our understanding of factors influencing synapse formation and TCB activity and provides a framework to guide dose optimization and therapeutic design to achieve a favorable benefit–risk profile. Translating this model to patient populations and validating it with clinical data will be essential to fully leverage its potential in supporting clinical decision-making. This includes tailoring dosing regimens to balance efficacy and safety, and prioritizing targets and indications for improved patient outcomes.

## Data Availability

The raw data supporting the conclusions of this article will be made available by the authors on request.

## Supplementary Materials

The following supporting information can be downloaded at: https://www.mdpi.com/article/10.3390/pharmaceutics17040500/s1, Figure S1: Workflow; Figure S2: Model simulation captures the distribution of exogenously administered active T-cells across blood and the different tissue types. Data digitized from Khot et al. 2019 [12]; Figure S3: Model prediction of steady-state resting T-cell tissue distributions; Table S1: List of parameters. Reference [47] is cited in the Supplementary Materials.
