# Correction: Susilo et al. Whole-Body Physiologically Based Pharmacokinetic Modeling Framework for Tissue Target Engagement of CD3 Bispecific Antibodies. *Pharmaceutics* 2025, *17*, 500

**DOI:** 10.3390/pharmaceutics18010012

**Published:** 2025-12-22

**Authors:** Monica E. Susilo, Stephan Schaller, Luis David Jiménez-Franco, Alexander Kulesza, Wilhelmus E. A. de Witte, Shang-Chiung Chen, C. Andrew Boswell, Danielle Mandikian, Chi-Chung Li

**Affiliations:** 1 Genentech, Inc., South San Francisco, CA 94080, USA; 2 ESQlabs GmbH, Am Sportplatz 7, 26683 Saterland, Germany

**Error in Figure**

In the original publication [1], there was a mistake in Figure 1 as published. An incorrect version of Figure 1 was included in the publication. This was purely a graphical error and does not affect the model or results. The figure legend for Figure 1 has been updated to rename the internalization rate constant (k_int_) as the elimination rate constant (k_elim_), given the definition of internalization which involves endocytosis. The corrected Figure 1 and its legend appear below:

**Text Correction**

There was an error in the original publication, where the parameters k_elim_ and k_int_ were incorrectly referenced in Section 2.3.

A correction has been made to Section 2.3, paragraph 3.

“Simulation scenarios for model calibration were defined according to the experimental setup in [9]. The CD3-related parameters (k_rec_, k_elim_, k_on_, k_off_) and HER2 parameters (k_syn_, k_deg_, k_elim_, k_on_, k_off_) were fitted in a hybrid, stepwise approach, using both manual adjustments and gradient-based parameter identification algorithms (Levenberg–Marquardt) included in the OSP Suite.”

The authors state that the scientific conclusions are unaffected. This correction was approved by the Academic Editor. The original publication has also been updated.

## Figures and Tables

**Figure 1:**
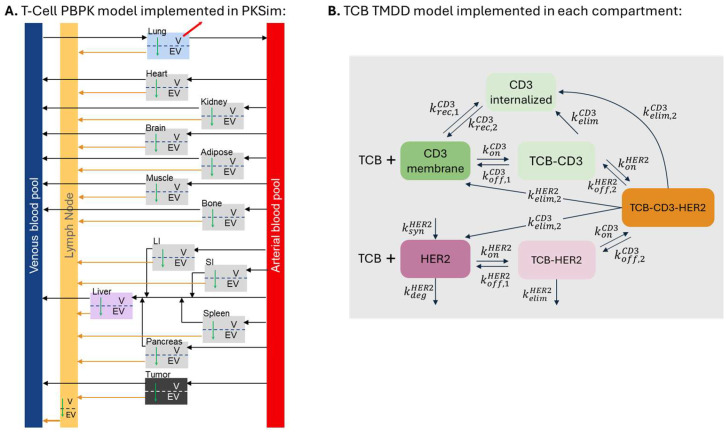
Schematic representation of (**A**) T-cell PBPK model and (**B**) T-cell-TCB-target cell synapse formation model. T-cell transmigration from vascular to extravascular space is indicated by the green arrow. k_syn_ refers to the zero-order target synthesis rate; k_deg_, k_rec_, and k_elim_ refer to the first-order degradation, recycling, and elimination rate contants of the target and the TCB-target complexes, respectively; k_off_ refers to the first-order TCB-target dissociation rate constants; and k_on_ refers to the second-order TCB-target association rate constants.

## References

[1] Susilo M.E., Schaller S., Jiménez-Franco L.D., Kulesza A., de Witte W.E.A., Chen S.-C., Boswell C.A., Mandikian D., Li C.-C. (2025). Whole-Body Physiologically Based Pharmacokinetic Modeling Framework for Tissue Target Engagement of CD3 Bispecific Antibodies. Pharmaceutics.

