# Supplementary material for: Whole-Body Physiologically Based Pharmacokinetic Modeling Framework for Tissue Target Engagement of CD3 Bispecific Antibodies
Source: Pharmaceutics. 2025 Apr 9;17(4):500. doi: 10.3390/pharmaceutics17040500 (PMC12030717; doi:10.3390/pharmaceutics17040500)
Supplement: Supplementary file 1 [file pharmaceutics-17-00500-s001.zip › pharmaceutics-3532389-supplementary.docx]

**Supplementary Materials**

Workflow


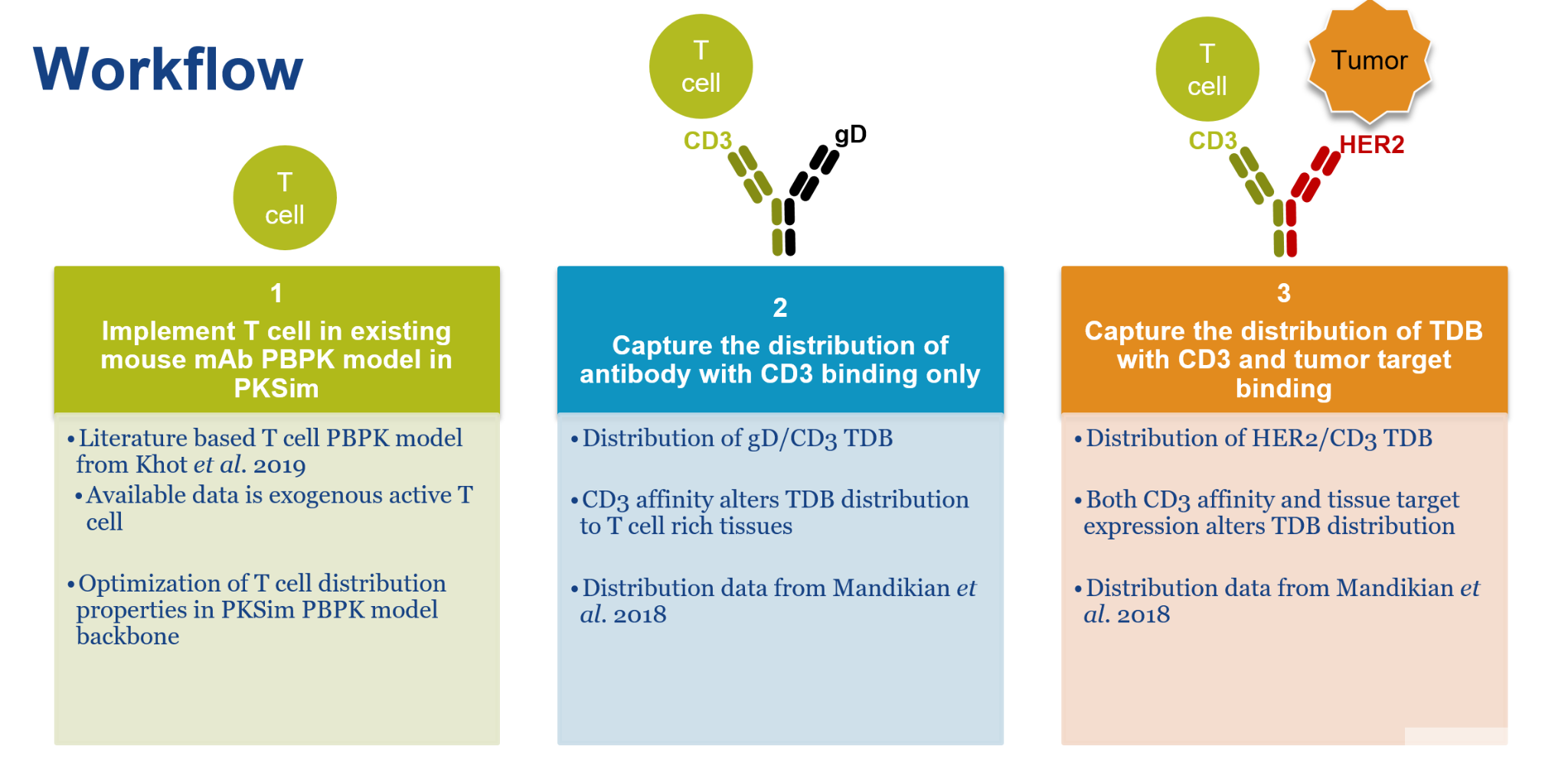


Supplementary Figure S1: Workflow

Details for the setup of PBPK model in PK-Sim

A Mouse PBPK Model with HER2 Expression has been created by generating an individual mouse with HER2 expression. For the sake of re-usability, HER2 has been renamed to “Target”. HER2 expression (while potentially informed on the whole organism level via PK-Sim database integration) was restricted to the tumor, with 31,811 per tumor cell = 356 µmol/L reference concentration, as non-tumor tissue had comparably negligible concentrations (Phillips et al. 2022).

The built-in large molecule module in PK-Sim was used, and six large molecules or “particles” representing different activated and naïve T-cells/CD3 states were defined. The model assumes proportionality of T-cell number and CD3. We chose to initialize CD3 concentrations proportional to a steady state T-cell level, where the distribution of CD3 represents the related T-cell distribution itself (setting the radius of each CD3 to 7 µm to avoid tissue passive distribution via the 2-pore model). To allow for several combinations of CD3 (membrane bound = m, internalized =i, bound to TCB = b) and T-cell states, we created six T-cell compounds in the PBPK modelTo define initial T-cell/CD3 concentrations, the model was run once into steady state with an initial IV bolus corresponding to the baseline native T-cell population and associate CD3 pool and reference concentration of 0.04 muM (distributed across binding/internalization states of CD3: (m,i,b=70:30:0).

The dedicated T-cell specific distribution processes for cells were implemented in MoBi to 1) describe the implemented T-cell transmigration from blood to the interstitium as a first-order rate equation and 2) the retention capability of T-cells in tissues to avoid drainage through lymph was implemented by a tissue-specific parameter to scale T-cells specific lymph drainage.

A new TCB molecule with a molecular weight of 150 kDa has been defined, including a protein binding partner for automatically creating the drug-receptor binding reactions (with HER2=“Target” ). The binding kinetics for TCB-CD3 and the CD§ and HER2 recycling for TMDD were then implemented in MoBi.

Results of the calibration of the T-cell model


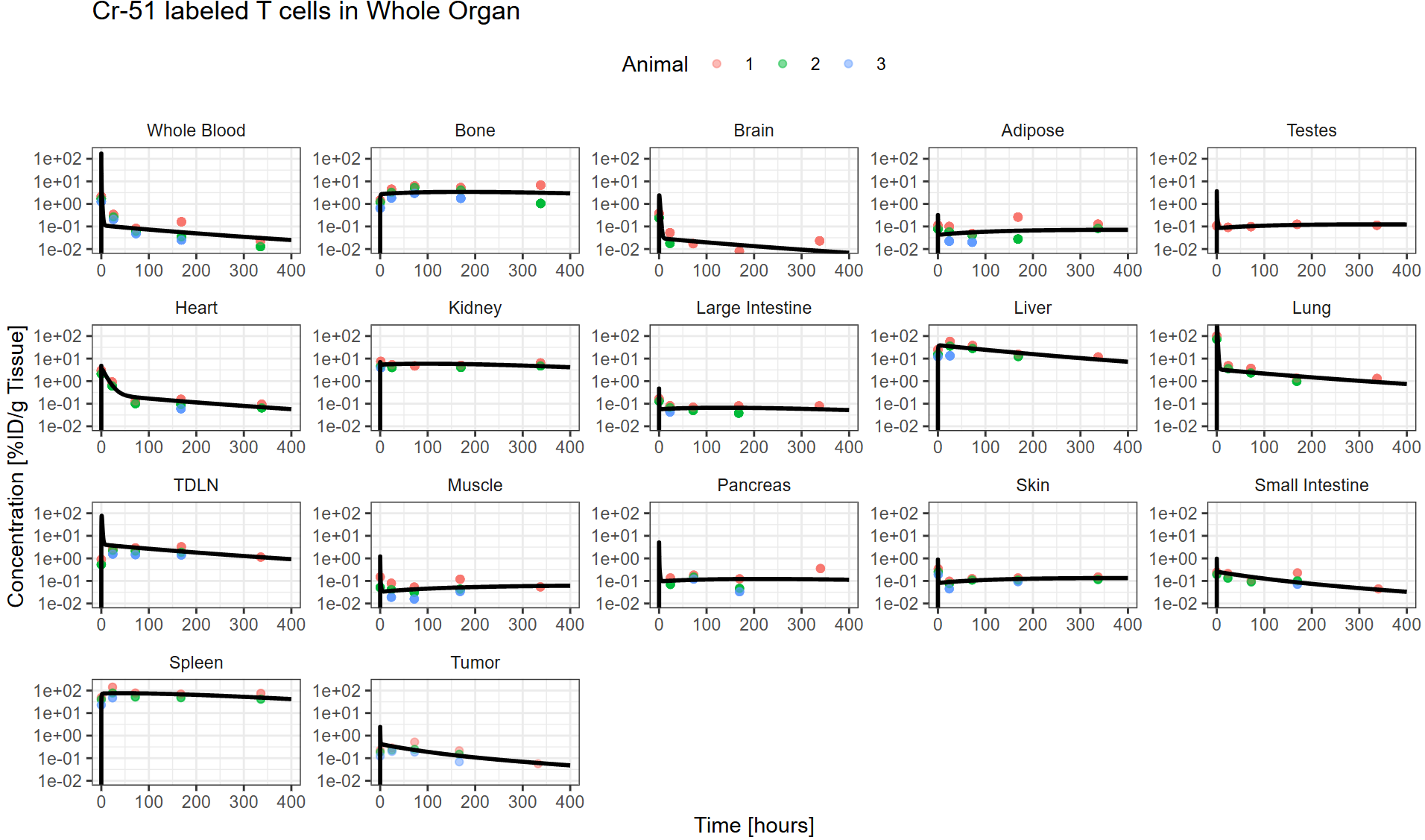


Supplementary Figure S2. Model simulation captures the distribution of exogenously administered active T cells across blood and the different tissue types. Data digitized from Khot et al. 2019 [(Khot et al. 2019)](https://paperpile.com/c/k0jtHU/xOr1)


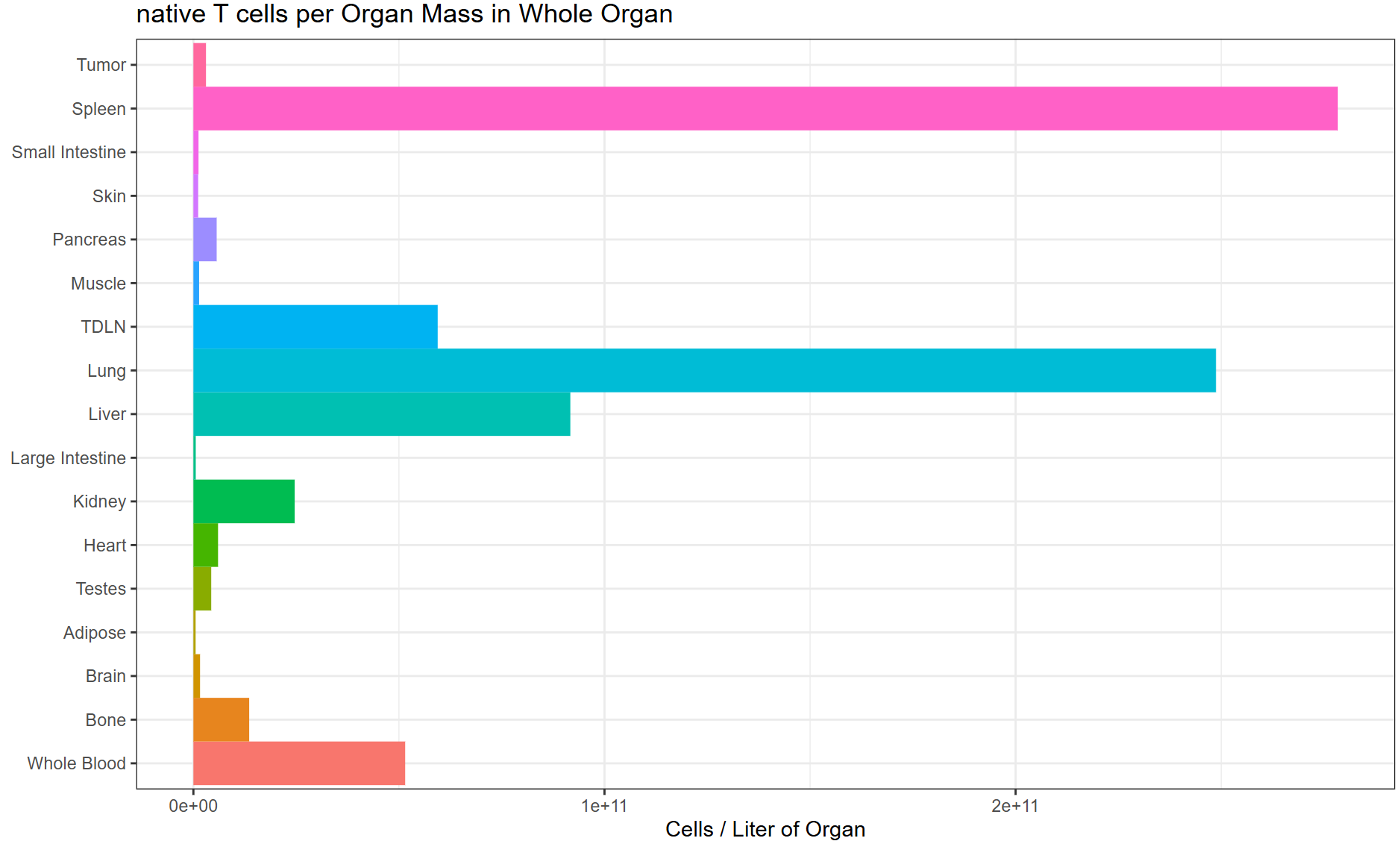


Supplementary Figure S3: Model-prediction of steady-state resting T-cell tissue distributions

**References:**

Lewis Phillips, G., Guo, J., Kiefer, J.R. *et al.* Trastuzumab does not bind rat or mouse ErbB2/neu: implications for selection of non-clinical safety models for trastuzumab-based therapeutics. *Breast Cancer Res Treat* **191**, 303–317 (2022). https://doi.org/10.1007/s10549-021-06427-w
